# Supplementary material for: Bona Fide Tumor Suppressor Genes Hypermethylated in Melanoma: A Narrative Review
Source: Int J Mol Sci. 2021 Oct 1;22(19):10674. doi: 10.3390/ijms221910674 (PMC8508892; doi:10.3390/ijms221910674)
Supplement: Supplementary file 1 [file ijms-22-10674-s001.zip › ijms-1377288-supplementary.pdf]

Supplementary table S1: bona fide tumor suppressor genes methylated or deleted in melanoma									
**TSG**	**Protein**	**Location**	**Protein role**	**Pathway**	**Hallmark of cancer**	**First author**	**Year**	**Sample type**	**Method**
PTEN	Phosphatase and tensin homolog	10q23.31	Multifunctional phosphatase, regulating apoptosis, regulating secretion of MMPs and IGFs and expression of VEGF	PTEN → PI3K↓ → AKT↓→ apoptosis	Apoptosis ↓	Mirmohammadsadegh A.	2006	Cell lines (Serum) (Tissue)	AKT kinase assay: 5-Aza → phosphorylation GSK3↓
					Tumor growth ↑ Metastasis ↑	Hwang P. H.	2001	Cell line Nude mice	1.PTEN-vector in cell line → colony formation↓ + no difference in proliferation 2.The above cell line in mice → tumor volume↓ + number of metastasis↓
					Tumor growth ↑	Inoue-Narita T.	2008	Mice	1. Mice with PTEN-deficient melanocytes → no spontaneous melanoma + larger melanocytes 2. The above mice + DMBA/TPA → large nevi and invasive spindle cell melanoma ↑
BRMS1	Breast cancer metastasis suppressor 1	11q13.2	Regulation of transcription, angiogenesis and other metastasis-related genes	/	Invasion ↑ Metastasis ↑	Shevde L. A.	2002	Cell lines	1. BRMS1 in cell lines without it → invasion↓ + no difference in cell morphology and primary tumor growth 2. The above cell lines in mice → metastasis↓
				BRMS1 → (ING4) → NF-κB (p50/65 subunits)↓ → IL-6↓ → angiogenesis↓	Angiogenesis ↑	Li J. (2 articles)	2010	Cell lines Nude mice	1. BRMS1/ING4 in cell lines → human umbilical vein endothelial cell growth↓ + tubular structures↓ 2. BRMS1/ING4 knockdown in cell lines → human umbilical

									vein endothelial cell growth↑ + tubular structures↑<br>3. The first cell lines in mice → angiogenesis↓
FERMT3<br>KIND3	Kindlin-3	11q13.1	Integrin activating protein	Kindlin-3 → Talin → integrins β1/3/5 → cell attachment	Migration ↑<br>Invasion ↑<br>Proliferation↑<br>Cell/tumor growth↑<br>Metastasis ↑	Djaafri I.	2014	Cell lines<br>Nude mice<br>(Tissue)	1. FERMT3 overexpression in cell lines → migration↓ + invasion↓<br>2. FERMTS knockdown in cell lines → migration↑ + invasion↑ + proliferation↑ + anchorage-independent growth↑<br>3. FERMT3 KO mice → tumor growth↑ + lung metastases↑
AKAP12<br>SSeCKS	A-kinase anchoring protein 12	6q25.1	Controlling the secretion of tumor chemo-attractants	AKAP12 → CXCL9/10↓ → metastatic chemotaxis↓	Metastasis ↑	Muramatsu M.	2017	Cell lines in transgenic mice	AKAP12 KO mice → peritoneal, liver and lung metastases↑ + no difference in primary tumor growth
CDKN2A	p16INK4a<br>p14Arf	9p21.3	1. p16INK4A: cell cycle regulation, oncogenic signaling, DNA damage, physiologic aging, inhibition of proliferation, senescence.<br>2. p14ARF: induction of ROS generation, cell cycle regulation	1. p16INK4 → CDK4/6↓ → cyclin D1 complex formation↓ → pRB1↓→ tumor growth through unlimited cell cycle progression↓<br>2. p16INK4 → CDK4/6↓ → cyclin D1 complex formation↓ → pRB1↓→ E2F1↓ → BRN2↓→ invasion↓<br>3. p14ARF → ROS generation → p53 activation → tumor cell growth/proliferation↓<br>4. p14ARF → complex wit h MDM2 → p53 degradation↓	Proliferation ↑<br>Tumor growth↑<br>Apoptosis↓<br>Migration↑<br>Invasion↑	Bai M.	2016	Cell line<br>Nude mice<br>(Tissue)	1. CDKN2A (P16INK4A or p14ARF) overexpression in cell line without it → proliferation↓ + cells in G0/G1↑ + apoptosis↑ + migration↓ + invasion↓<br>2. The above cell line in nude mice → tumor growth↓
					Proliferation	Zeng H.	2018	Cell lines	1. CDKN2A null NHMs:

					↑<br>Invasion ↑<br>Motility ↑<br>Migration ↑<br>Metastasis ↑			Nude mice	proliferation↑ + motility↑(independent of driver mutation and proliferation) + migration↑ + invasion↑<br>2. Cell lines in nude mice → metastasis↑ in CDKN2A knockdown cell line + metastasis↓ in p16INK4a expressing cell line (without correlation with proliferation)
					Proliferation ↑<br>Tumor growth↑<br>Senescence ↓	Regneri J.	2019	Xiphophorus melanoma cell line<br><br>Medaka fish	1. Cdkn2ab (fish ortholog) overexpression in cell line → proliferation↓ + senescence-like phenotype<br>2. In vivo co-expression of Xiphophorus cdkn2ab in medaka with oncogene and its promotor → melanoma development↓<br>3. In vivo CDKN2A KO medaka with oncogene and its promotor → tumor growth↑
APAF-1	Apoptotic peptidase activating factor 1	12q23.1	Mediating p53-dependent apoptosis	p53 → Bax ↑ → cytochrome c ↑ → Apaf-1/Casp9 death effector complex	Apoptosis ↓	Soengas M. S.	2001	Cell lines (Tissue)	Retroviral APAF-1 transfer/5-Aza in cell lines → apoptosis↑ + adriamycin (p53 activator) sensitivity↑
MTAP	Methylthio-adenosine	9p21.3	Catalyzation of the phos- phorylation of	MTAP↓ → MTA accumulates (inhibition of methyltransferases) →	Invasion ↑	Behrmann I.	2003	Cell lines (Tissue)	MTAP re-expression in cell line → invasion↓ + no

	phosphorylase		methyl-thioadenosine (MTA)	methylation of arg31 in STAT1 by PRMT1↓ → binding PIAS to STAT1 → inhibition of STAT1 DNA binding activity → interferon-ⲁ and -β signaling pathway↓ → IFN-treatment response↓					change in proliferation
MEN1	Menin 1	11q13.1	Role in transcription, cell cycle regulation and proliferation, oncogene induced senescence in melanocytes, maintenance of genome integrity.	DNA damage → ATM/ATR →MEN1 phosphorylation (stabilization) → MEN1 + MLL → ESR1 → BRCA1, RAD51, RAD51AP1 → H3K4me3↑ → transcription of target genes (HOX, CDKI)	DNA repair ↓ Cell growth ↑	Fang M.	2013	Cell lines	1. MEN1 overexpression in cell line → growth ↓ 2. MEN1 knockdown in cell line → double-stranded DNA breaks ↑ + homologous recombination repair ↓ + nonhomologous end-joining repair ↑
